# Failing Is Derailing: The Underperformance as a Stressor Model

**DOI:** 10.3389/fpsyg.2020.01617

**Published:** 2020-07-16

**Authors:** Shani Pindek

**Affiliations:** Department of Human Services, University of Haifa, Haifa, Israel

**Keywords:** performance, underperformance, stress, strain, consequence of error, medical error, self-efficacy

## Abstract

Job performance and job stress are widely studied phenomena in occupational research. However, most literatures on the relationship between work stress and job performance conceptualize job stress as an antecedent of performance, in line with the stress-performance framework, and do not examine what happens to the well-being of the employees after the performance was appraised as poor. In the current theoretical paper, I argue that task underperformance is a source of stress (i.e., stressor) for the employee and, as such, can affect a wide range of employee outcomes. Task underperformance is conceptualized as comprised of two main types: acute/episodic underperformance, such as a mistake or an accident (e.g., medical error and service failure), and chronic task underperformance, such as not achieving the expected work products over time, with an interplay between these types. The source of the appraisal (objective, supervisor-rated, and self-rated underperformance) is also considered. Several disjoint literatures are then integrated in order to explain how underperformance is expected to result in subsequent decrements to employee well-being. At the chronic underperformance level, the following literatures are included: self-efficacy, negative effects of performance feedback, and stress experienced when the basic need for competency is frustrated or when underperformance presents a threat to the self-image. At the acute/episodic level, affective and cognitive outcomes are explored, and examples are drawn from several industries including service failures and medical errors. The interplay between the two types of underperformance, acute/episodic and chronic, is discussed, and then relevant moderators are offered. One notable moderator is the occupation-level consequences of error, which likely affects most if not all outcomes. Finally, the discussion includes potential theoretical and practical implications for this conceptualization, as well as some methodological considerations for future research in this area.

## Introduction

**Figure 1:**
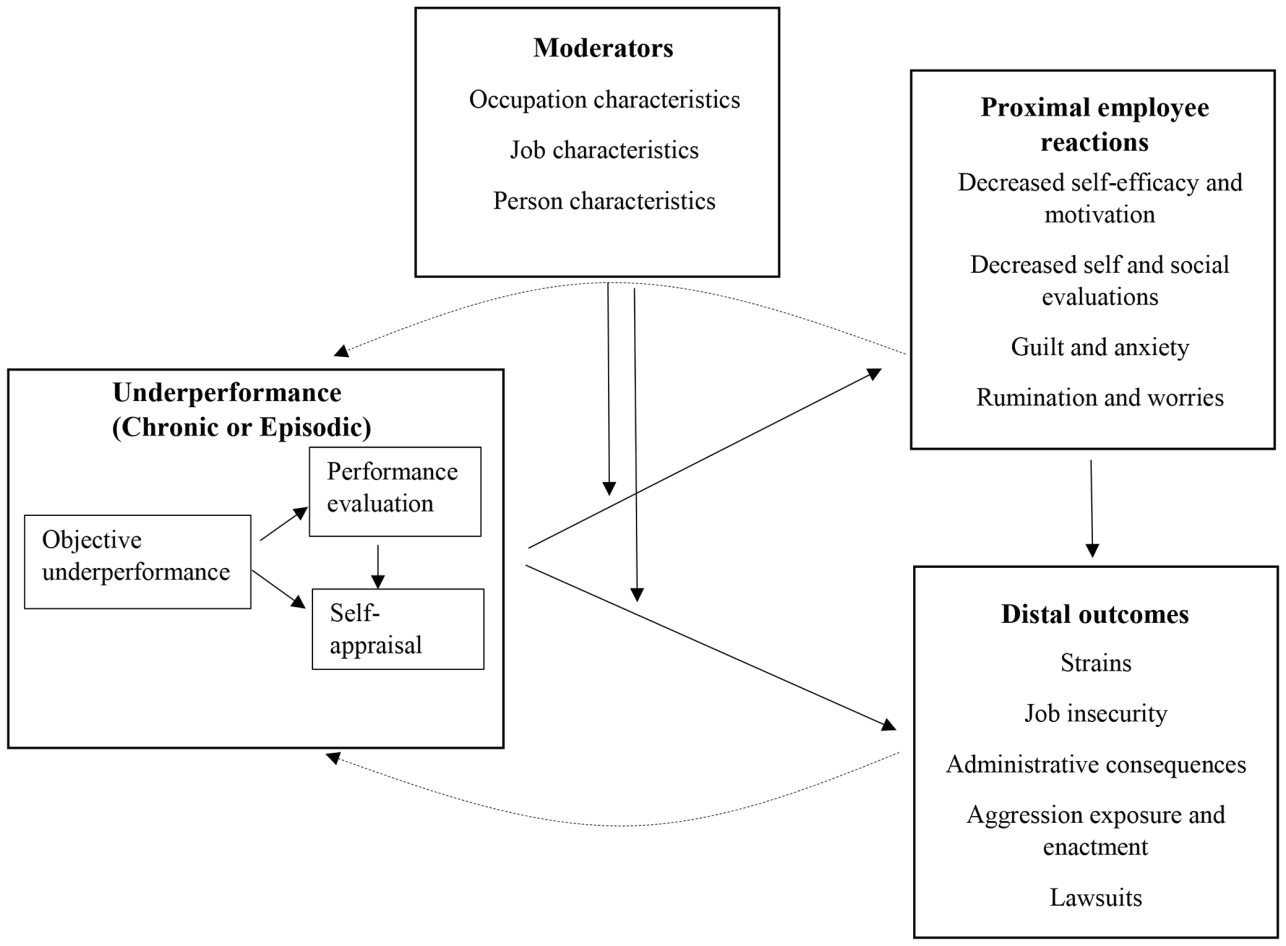
The overall underperformance as a stressor model. The dashed lines indicate the traditional direction of effects from employee stress to employee performance, while the solid lines present the direction of effects conceptualized in the current paper.

Job performance and job stress are two of the most studied phenomena in occupational research, with more than 40 meta-analyses examining relationships between variables from these two domains (Pindek et al., 2017). However, most literatures on the relationship between work stress and job performance conceptualize job stress as an antecedent of performance, in line with the stress-performance framework (Jex, 1998; Bliese et al., 2017) and do not examine what happens to employees in the aftermath of poor performance. In the current conceptual paper, I argue that task underperformance, whether objective, supervisor-rated, or self-rated, is a source of stress (i.e., stressor) for the employee. As such, underperformance can affect a wide range of employee outcomes, including subsequent decrements to self-perceptions, physical and mental well-being, and motivation, as well as increased intention to turnover. These outcomes, while potentially predictive of further decrements to performance over time (Lindsley et al., 1995), are also worthwhile examining in and of themselves, keeping the well-being of the employee, not just the organization, at the center of attention. The overall underperformance model, which will be developed in this theoretical paper, is presented in Figure 1.

This review makes four primary contributions to our understanding of the effects of underperformance on the well-being of employees. First, underperformance is reconceptualized as source of stress for the employee, distinct from other performance-related stressors. Examining underperformance as a source of stress is not meant to discount the established literature on the link between employee stress and its effects of employee performance. This traditional path is demonstrated by the dashed line in Figure 1. Rather, it is meant to emphasize a commonly forgotten component of this process, which is that underperformance has well-being consequences for the employee. Second, underperformance is conceptualized as a stressor with two main types: acute/episodic, and chronic. These two types are distinct and can have different consequences for the employees. Third, several disjoint literature streams are integrated in order to support predictions pertaining to prominent outcomes and moderators. Some of these are unique to specific types of underperformance or to specific jobs, and others are relevant across most jobs. This integration increases generalizability and enables better understanding of the consequences of underperformance across occupations. Fourth, practical research considerations are discussed. These can serve as an initial guideline for researchers who are interested in understanding the effects of underperformance. The practical implications can be of use to practitioners who wish to better care for their employees when they are underperforming. Throughout this paper, testable propositions are presented with the overall purpose of igniting research using this nontraditional paradigm that focuses on the well-being of underperforming employees.

### Definition the Two Types of Underperformance

First, it is important to define underperformance, and specifically task underperformance, as it pertains to employee well-being. The two underperformance types are acute/episodic underperformance, such as a mistake or an accident (e.g., medical error, service failure), and chronic task underperformance, such as not achieving the expected work products over time. *Acute/episodic underperformance* is limited in time, typically to a specific task that is being carried out. It is defined here as carrying out a task in a way that does not meet the standards for performance or that results in outcomes that are noticeably worse than they could have been if a different option of carrying out the task had been chosen. This type of underperformance varies greatly among occupations, in terms of both frequency and severity of underperformance episodes as will be reviewed later. *Chronic underperformance* is defined as not meeting the standards for effectiveness requirements in terms of quality and/or quantity of the output that the employee has produced over time. This definition is based on existing definitions of task performance (e.g., Rotundo and Sackett, 2002; Gilboa et al., 2008). Underperformance refers to actions that are under the control of the employee, in the sense that external factors that influenced outcomes are not considered part of the performance appraisal. However, an innate ability is included in performance appraisals even though it may be beyond the employee’s control.

In this paper, the focus is on the stress effects that are experienced by the underperforming employee. The stressful experience of having co-workers, subordinates, or even leaders who are underperforming, while important, are beyond the scope of this conceptualization.

The two types of underperformance are distinct: both chronic high performers as well as chronic underperformers can exhibit instances of acute/episodic underperformance. Furthermore, an employee who is generally an average performer can have fewer instances of acute/episodic underperformance than a generally high performer. This can happen, for example, if the average performing employee tends to take on tasks that are less complicated or error-prone than the generally high performing employee. It is, therefore, proposed that chronic and acute/episodic underperformance can have different meanings and consequences.

### Sources of Underperformance Ratings

Another relevant distinction has to do with the source of the underperformance ratings. This conceptualization considers underperformance when the performance level is (a) objectively measured, such as when a sales employee fails to meet sales goals or when a medical error occurs; (b) appraised by the supervisor, such as periodic performance appraisals; or (c) self-appraised. The different perspectives on the same person’s performance level, or sources of the ratings, likely affect the range of possible consequences. As shown in the first part of Figure 1, objective underperformance will likely also be reflected in both supervisor-rated and self-rated underperformance. Furthermore, supervisor-rated underperformance is usually communicated to the employee and can be reflected in the employee’s self-ratings as well. Therefore, objective and supervisor-rated underperformance can affect outcomes both directly and *via* self-rated underperformance. Consequently, we can expect many similarities in the effects that underperformance has on employee’s well-being across the different sources. However, it is important to remember that there are also many additional factors that influence subjective performance ratings (supervisor-rated or self-rated) that are beyond the objective performance level (e.g., Klimoski and London, 1974). These are beyond the scope of the current paper, as the focus here is on the effects of the source of ratings on outcomes rather than factors that influence the ratings themselves.

When underperformance is objective or supervisor-rated, and is known not only to the employee but also to the organization or the manager, there can be additional outcomes, such as increased job insecurity and potential layoffs. For example, if an employee made a medical error and a patient died, this episodic underperformance is likely known to the organization and to the patient’s family and may result in a lawsuit and an increased risk of termination. However, if a medical decision was made that is in line with care practices but is not optimal, or if there was a near-miss, it is possible that only the employee will be aware of this milder underperformance, likely leading to consequences such as lower self-perceptions and decrements to well-being without the increased risk of a lawsuit or termination.

The current conceptualization focuses on the effects of underperformance as a stressor for the individual and not for the organization. The transactional model of stress is therefore applicable in explaining how a stressor must at first be perceived and appraised as a threat (Lazarus and Folkman, 1984), and only then a stress response can occur. Consequently, underperformance that the employee is not aware of (e.g., a doctor made a medical error but that error was not immediately apparent and the doctor was never made aware of it) is not likely to elicit a stress response. However, we can expect that when the employee is aware of his/her underperformance, the source of the rating affects the type of outcomes it will have, with objective or supervisor-rated underperformance both affecting self-rated underperformance and holding additional consequences for the employee beyond self-rated underperformance.

***Proposition 1**: The effects of underperformance on employee reactions and outcomes depend on the source of underperformance appraisal (objective, supervisor appraisals, and self-appraisals)*.

### Distinctions From Other Performance-Related Stressors

A theoretical distinction must be made between underperformance as a stressor and other performance-related stressors. Underperformance is different from organizational constraints (i.e., aspects of the work environment that interfere with effective performance) because organizational constraints are considered external factors, beyond the employee’s control (Pindek and Spector, 2016b), while underperformance is focused on internal factors such as ability and effort. Furthermore, underperformance is a level of performance, and organizational constraints have only a weak association with performance levels (Pindek and Spector, 2016a).

Another performance related stressor that is relevant here is performance pressure, defined as a mindset focused on the necessity for high performance (Eisenberger and Aselage, 2009). This mindset is often influenced by the organizational culture. Underperformance is different from pressure to perform because the latter deals with a demand for high performance (usually in terms of volume and quality) and examines performance-related outcomes (as well as some strains) of that demand, while the current conceptualization is of appraisals of underperformance that has already transpired, regardless of performance pressure. Performance pressure is associated with one central positive outcome, higher intrinsic interest (Eisenberger and Aselage, 2009), though it can lead to some negative consequences as well (e.g., Gardner, 2012). Underperformance, on the other hand, is conceptualized as a source of stress for the employee that is typically associated with lower self-efficacy and, only under some circumstances, will result in an increased effort aimed at improving future performance. Another difference is that performance pressure is focused on the high end of the performance scale, while underperformance is focused on the low end. Therefore, these two performance-related stressors, organizational constraints and performance pressure, are theoretically distinct from underperformance.

***Proposition 2**: Underperformance is distinct from organizational constraints and performance pressure*.

## Theoretical Background

In order to conceptualize underperformance as a stressor that can result in subsequent decrements to employee well-being, several disjoint literatures are integrated for each of the two types: acute/episodic and chronic underperformance. Empirical evidence for the two types is often intermixed, and they share some (but not all) proximal and distal outcomes. Proximal outcomes include reduced self-efficacy, decreased self and social evaluations, negative emotions (e.g., anxiety, guilt/shame, and sadness), and cognitions (e.g., self-esteem and rumination). Distal outcomes are delayed or have longer term responses such as negative behaviors (e.g., poor health behaviors and withdrawal behaviors), as well as employment-related outcomes (e.g., higher risk of termination or demotion and lawsuits). These are shown in Figure 1 and discussed in the following sections.

One important note is that much of the research that is reviewed in this paper, including both primary studies and meta-analyses, relied predominantly on cross-sectional designs. The data gathered in these studies that is often meant to support one hypothesized direction of effects (e.g., effects of self-efficacy or emotions on employee performance) do not in fact support any one direction of effects, but only the association between two phenomena. For example, a meta-analysis of cross-sectional effects between emotions and performance (Shockley et al., 2012) only supports the connection between the two phenomena and cannot distinguish between the hypothesized direction in the meta-analysis, whereby negative emotions lead to poorer performance, and the reverse direction, proposed in the current paper, whereby underperformance leads to negative emotions. Most of the hypotheses raised in the current paper are based on theoretical development, and the provided evidence is only for the link between the phenomena. The needed supporting evidence for the proposed direction of effects is still lacking in most cases. This is discussed further in the methodological consideration for future research.

### Chronic Underperformance as Stressor

Several related psychological constructs and theories have been developed over the years that can be used to explain why chronic underperforming is a stressor for employees, and can therefore be expected to result in strains or decrements to well-being, in line with the stressor-strain framework (Jex and Beehr, 1991). The suggested proximal employee responses rely on the self-efficacy and feedback literatures that are focused ultimately on continued task performance. However, these literatures also discuss well-being indicators and are therefore useful for the current conceptualization. Two additional theories, basic psychological need theory and stress-as-offense-to-self theory, are used to explain stain outcomes of underperformance. In the last part of the current section, more distal outcomes are reviewed, and supporting empirical evidence is presented.

#### Chronic Underperformance: Proximal Employee Reactions

Self-efficacy is a person’s belief in his/her ability to accomplish or master a task (Bandura, 1986). The basic mechanism underlying the relationship between self-efficacy and performance is that when one believes in one’s ability to accomplish a goal, that person will invest sufficient effort and will more likely achieve the goal. This achievement in turn confirms the person’s initial efficacy beliefs and boosts the self-efficacy level further, constituting a positive efficacy-performance spiral (Lindsley et al., 1995; Shea and Howell, 2000). The opposite pattern of a negative spiral has also been discussed: decreased efficacy and decreased performance are part of a negative loop, whereby the decreased performance is attributed to the self, and the underperformance becomes more internalized and stable over time (Lindsley et al., 1995). According to this perspective, underperformance can, over time, result in a stable assessment that an employee makes about his/her own ability to meet the performance goals. When the employee assesses himself/herself as having low ability to achieve the goal, then the employee will likely invest lower efforts as well as exhibit greater avoidance and withdrawal.

Another related construct is that of feedback. Much of the self-efficacy literature discusses high-quality performance feedback as a means of avoiding negative self-efficacy spirals. That is, while information on the performance outcome (failure/success) is a crucial building block of efficacy-performance spirals, high quality feedback is considered a key to self-correction and a way out of a negative spiral (Lindsley et al., 1995). When feedback that contains information on a discrepancy between the previous task performance and the desired standards for performance is given, a self-related discrepancy arises whereby the individual’s self-view is not meeting one’s own standards for success. This self-related discrepancy can result in an increased engagement in the task in order to meet the performance standard and reduce the discrepancy. However, it can also result in a shift of focus away from the task, because redirecting one’s efforts on a different activity where a positive self-view is more easily attainable is another way of reducing the self-related discrepancy (Kluger and DeNisi, 1996). This process is again dependent on the attributions made by the employee: if underperformance is attributed to lack of sufficient effort (external and changeable), an increased engagement in the task is more likely. However, if underperformance is attributed to lack of ability (internal and stable), then decreased engagement is more likely, as well as a host of negative outcomes to the well-being of the employee (Silver et al., 1995; Ilgen and Davis, 2000). Therefore, these literatures support an association between stable and chronic underperformance and decreased engagement and self-efficacy.

Both the self-efficacy and the feedback streams of literature were focused on task performance as the ultimate outcome variable, and looped decreased performance, *via* reduced effort or *via* feedback and resulting anxiety, to future decreased performance. In the current conceptualization, the focus is on just one part of the process, the association between underperformance and the resulting decrease in self-efficacy. However, this largely disregards other outcomes of the anxiety associated with underperformance, which are well-known in the occupational health literature. Early studies have shown a correlation between self-efficacy and both frustration and anxiety (Jex and Gudanowski, 1992), and other studies found that self-efficacy is associated with mental well-being of employees (as well as physical well-being, though to a lower extent), as well as buffers the relationship between stressors and mental and physical well-being (Jex and Bliese, 1999; Siu et al., 2007). Similarly, Avey et al. (2009) discussed how having greater psychological capital (efficacy, optimism, and resilience) will be negatively associated with stress and intentions to quit. Therefore, it is likely that these proximal employee reactions of decreased efficacy and motivation can lead to additional anxiety based well-being outcomes for employees.

***Proposition 3**: General underperformance leads to decreased self-efficacy and motivation and increased anxiety*.

Other psychological theories provide a more direct framework for examining the negative outcomes of underperformance to employees, because their focus is on the well-being of employees rather than on their future performance. The need for competence, one of the three basic needs described in the Basic Psychological Need Theory (BPNT; Deci and Ryan, 2000; Ryan and Deci, 2000), refers to the need to experience efficacy and mastery when coping with stimuli from the work environment (Deci and Ryan, 2000). The need for competence is conceptualized as a fundamental need. As such, the frustration of this need that is apparent when an individual underperforms over time (thus likely attributing the underperformance to a stable cause) would relate directly to feelings of failure and doubts about one’s own efficacy. If there is an external constraint, or reason for underperforming, the individual might make an external attribution, protecting their self-efficacy at the expense of their external efficacy (defined as “the belief in the utility of the means available for performing the job”; Eden, 2001, p. 74). While this can protect the individual’s self-esteem to some extent, the need for competence is still frustrated. The frustration of this basic need can result in decrements to various well-being indicators, such as physical health, depressive symptoms, life satisfaction, and vitality (Chen et al., 2015; Kim and Allan, 2019). A related theory that builds on goals and needs frustration is the Stress-as-Offense-to-Self (SOS) theory (Semmer et al., 2007, 2019). According to this theory, people strive to maintain a positive self-image, and threats to this image result in the experience of stress. For many people, a large part of their identity is tied to their work (Ashforth et al., 2008). Failing to reach performance standards may threaten the self because competency in a domain that is tied to the individual’s identity is a basic need (as it is the basis for self-esteem related to professional identities). This threat to the self is affected by both self-evaluations and evaluation by others. Specifically, when underperformance is known to others (such as when it is appraised by the supervisor), on top of reduced self-esteem, it can also be associated with reduced social validation, which signals a movement further away from the individual’s desired social self (Ashforth and Schinoff, 2016). These decreases to self and social evaluations are therefore additional proximal responses for chronic underperformance, which can have more distal consequences of their own.

***Proposition 4**: Chronic underperformance leads to decreased self and social evaluations*.

#### Chronic Underperformance: Distal Outcomes

As with any stressor, underperformance is expected to have many well-being outcomes. Imperative to this discussion of potential outcomes is the source of underperformance ratings that was discussed above. Self-rated underperformance can originate from objective underperformance or from receiving poor performance ratings by the supervisor. If the employee agrees with the assessment of underperformance, and judges him/herself as performing poorly, or if the self-rated underperformance is independent of these external sources, then based on this conceptualization of underperformance as a stressor, many strain (i.e., health and well-being outcomes of stressors) outcomes can be expected, as part of a stressor-strain process (Spector and Fox, 2002) whereby first an immediate affective response occurs, and then more distal strains follow.

In addition to the proximal self-efficacy, motivation, and self-esteem mechanisms described above, chronic underperformance is associated with many types of negative outcomes, though evidence is based on cross-sectional correlations. For example, underperformance is associated with affective strains, such as anxiety and sadness (but not anger or frustration), and the relationship is thought to be reciprocal, whereby negative effect results in decrements to performance, and underperformance results in negative effect (Shockley et al., 2012). Furthermore, cognitive outcomes can also be expected. Studies often report that cognitive rumination impairs consequent performance (Baranik et al., 2014), but the very definition of rumination is recurring thoughts that revolve around an instrumental theme of failure to achieve a goal (Martin and Tesser, 2006). Therefore, underperformance likely results in rumination. These affective and cognitive outcomes are, perhaps, more relevant to the discussion on acute/episodic underperformance. This is because while many of these studies on affective and cognitive outcomes use designs that capture person-level summary judgments of performance levels, in line with chronic underperformance, their theoretical underpinnings are better suited to explain a negative effect or cognitive rumination following a specific negative event such as acute/episodic underperformance. Nevertheless, in their discussion on the meta-analytic findings, Shockley et al. (2012) note that cumulative episodes of state sadness or anxiety may result in long-term depression or mood disorders, which can have further effects on health, absenteeism, and future underperformance (Berto et al., 2000; Kessler et al., 2006; Plaisier et al., 2010). Similarly, habitually ruminating on negative work experiences such as underperforming is associated with heavy alcohol use, workday alcohol use, and after work alcohol use (Frone, 2015). This increased alcohol use has long-term detrimental health consequences for the employee, and shorter-term detrimental health consequences for the employee’s continued ability to achieve performance goals, particularly when engaging in workday alcohol use.

A large scale cross-sectional study found that low self-rated performance is associated with additional poor health behaviors, such as poorer eating habits, in addition to poorer physical and mental well-being, including depression, asthma, and heart attacks, to name a few (Merrill et al., 2013). These health behaviors and mental and physical well-being variables are likely part of a cyclic relationship with performance, because while chronic underperformers can experience physical and mental strain as an outcome *via* the typical stressor-strain process (Spector and Fox, 2002), their poorer health can also keep them from hitting their performance goals (Merrill et al., 2013). In summary, chronic underperformance can have various negative outcomes, similar to most other stressors.

***Proposition 5**: Chronic underperformance leads to negative mental, physical, and behavioral strain outcomes via a stressor-strain process*.

In addition to the typical strain outcomes characteristic of many stressor-strain relationships, when dealing with objective underperformance, or supervisor-rated underperformance, there can be additional potential consequences that pertain to the administrative purposes of performance appraisal. Examples for such administrative consequences include not getting a pay raise, getting fired, or being demoted to a lower-level job. Even if these administrative steps are not taken, there will likely be an increased sense of job insecurity, which is known to adversely impact employee well-being (Dekker and Schaufeli, 1995; Sverke et al., 2002; Jiang and Lavaysse, 2018). Furthermore, if the employee disagrees with the performance appraisal of his/her supervisor, the employee might perceive the organization as unjust or perceive a poor fit with the organization or the job. Both perceived injustice and poor fit are likely to result in anger and an increased intention to turnover (Cohen-Charash and Spector, 2001; Verquer et al., 2003).

One cross-sectional study using a large heterogeneous sample (Schreurs et al., 2010) found a negative association between job insecurity and self-efficacy. Other studies have found negative associations between job insecurity and self-rated (Staufenbiel and König, 2010) as well as supervisor rated (Wang et al., 2015) job performance. Though the direction of effects was not directly tested, it is theoretically plausible that underperforming over time results in decreased self-efficacy, which then results in greater job-insecurity. Furthermore, both job insecurity and decreased self-efficacy were associated with poorer general health of the employees (Schreurs et al., 2010), in line with the idea of a cascade of negative outcomes following underperformance. Job insecurity is a serious concern for the health and well-being of employees, as it is associated with a whole host of negative consequences, ranging from poorer job satisfaction and optimism about the future, to poorer physical and mental health, and even some changes in personality (Wu et al., 2020).

A related outcome is job termination. When a person is laid off, a multitude of negative outcomes can arise, including poorer mental health, career outcomes, and even an increased suicide risk (Classen and Dunn, 2012). Job loss is associated with increased risk of mortality in different ways beyond increased suicide risk, including higher rates of certain diseases (e.g., cardiovascular disease) and unhealthy behaviors, such as unsafe driving leading to more traffic accidents and increased alcohol consumption leading to more alcohol-related disease (Browning and Heinesen, 2012). The general greater chance of dying in the years following the job loss is thought to increase by a substantial amount, implying a reduction of 1–1.5 years in life expectancy for an employee who lost a job at the age of 40 (Sullivan and von Wachter, 2009). Although causality is hard to determine here, because it is possible that underlying conditions caused both the underperformance and the subsequent decrease in physical well-being, it is reasonable that job insecurity and termination are potential outcomes for chronic underperformance. These phenomena are important because they can potentially affect employee well-being to a great extent.

***Proposition 6**: General underperformance leads to job insecurity and increased chances of job termination*.

### Acute/Episodic Underperformance as a Stressor

Acute/episodic underperformance is not necessarily an indicator of general poor performance and can be associated with improved subsequent performance if learning occurs. This is the basic tenet of error management theory (Edmondson, 2004) that refers to the strategies in place that can reduce the future errors by learning from current errors. However, error management theory is only concerned with leveraging errors for improved future performance, and only recently (King and Beehr, 2017), researchers have begun focusing on the complex impact that making a mistake can have on the employee’s well-being. King and Beehr (2017) used the transactional theory of stress (Lazarus and Folkman, 1984) to explain how errors may lead employees to experience emotional strain, depending on how those errors are appraised. This theory consists of two stages of appraisal: primary appraisal is focused on the degree of harm or threat in the situation, and secondary appraisal is focused on the person’s perceived ability to cope with the situation that has been deemed threatening or harmful in the first stage. Within the context of acute/episodic underperformance, the severity of the consequences of an error will likely determine the primary appraisal of the situation as stressful or not. Many errors are inconsequential enough that they would not be appraised as harmful or threatening and would not result in stress. However, if acute/episodic underperformance is appraised by the employee as severe enough, then several proximal responses will likely follow, especially when secondary appraisal of ability to cope is low.

#### Acute/Episodic Underperformance: Proximal Employee Reactions

Some of the proximal employee reactions to acute/episodic underperformance are similar to the reactions to chronic underperformance, namely reduced self-efficacy and threats to self-worth. However, because acute/episodic underperformance occurs within a limited timeframe, it is also appropriate to more thoroughly discuss transient responses such as immediate affective responses (e.g., guilt and anxiety), and cognitive processes (e.g., rumination and worries). I describe the affective and cognitive responses separately, though the distinction between affect and cognition is often blurry. For example, the affective experience of anxiety and cognitive worries are often intermixed.

Affective responses include several distinct emotions that can arise in response to perceiving that one has made an error and that have known cross-sectional associations with performance levels (Brown et al., 2005; Shockley et al., 2012). Anxiety, for example, can be associated with anticipation of some form of retaliation against the employee who erred, including reduced compensation (e.g., in a sales job), potential lawsuit (e.g., in the case of a medical errors), or other administrative consequence (e.g., denied promotion, demotion, or termination). Anxiety is thought to intensify the focus on the unmet goals of the self after experiencing a negative performance outcome (Kluger and DeNisi, 1996). Therefore, individuals who experience anxiety are less likely to be able to leverage the episodic underperformance as a learning opportunity, and more likely to experience additional negative outcomes following an error that was made on the job.

Another affective response is guilt or shame. Episodic underperformance can be associated with a loss in self-esteem as well as a loss of regard by others (Brown et al., 2005; Sirriyeh et al., 2010). This loss of reputation and external judgments of incompetency is associated with shame (Wu, 2000; Delbanco and Bell, 2007; Daniels and Robinson, 2019). Guilt can therefore be an immediate reaction to a mistake that caused harm to another (e.g., a patient), and accompanying shame will be felt, particularly when this mistake is known to others. When feelings of guilt or shame are not dealt with appropriately in the short term, they can over time result in increased burnout and unhealthy coping behaviors such as alcohol and drug use (Wu, 2000). Sadness and disappointment may also arise in response to episodic underperformance.

The Shockley et al. (2012) meta-analysis included an account of the relationship between emotions and performance based on cross-sectional within-person designs, which are a better indication of episodic underperformance than of chronic underperformance. This within-person analysis revealed similar results to the between-person analysis, with an overall association between negative emotions and poorer performance, and some discrete emotions (i.e., sadness), but not others (e.g., anger), showing this effect. However, it is possible that different study designs would reveal additional emotional responses, such as frustration or anger with perceived external causes that contributed to the employee’s underperformance (e.g., organizational constraints). The cross-sectional results reported above are generally in line with the arguments made here, bearing in mind that they equally support both directions of effects between negative emotions and underperformance.

***Proposition 7**: Acute/episodic underperformance leads to negative emotions including anxiety and guilt/shame*.

The cognitive responses, rumination and worries, are two forms of perseverative thoughts. Rumination is past-oriented and entails repetitive thinking on what has already transpired, often with the hope of gaining insight into the meaning of or reasons for negative events (Nolen-Hoeksema et al., 2008). Worries are future-oriented and focus on anticipated threats. Work-related rumination and worries also have distinct motivational properties: the conscious motive underlying worries is to anticipate and prepare for future threats, whereas the conscious motive underlying rumination is to gain insight into the meaning of past negative events (Nolen-Hoeksema et al., 2008). The growing literature on mindfulness as a tool to buffer perseverative thoughts and thus reduce strain responses (e.g., Goldhagen et al., 2015) points to the centrality of these cognitive reactions. That is, when individuals are focused on their past behaviors or worried about a future threat in a judgmental way, the discrepancy between their ideals or expectations and the reality of underperforming results in strain experiences. This, like the negative affective responses, can have additional, more distal, strain outcomes.

In the context of underperformance, ruminating about an episode where the employee had made a mistake, or did not do his/her best can lead to a multitude of negative outcomes, such as sleep impairment, reduced well-being, and burnout (Wendsche and Lohmann-Haislah, 2017). Similarly, Pereira et al. (2013) found that work-related worries are related to subsequent sleep fragmentation, likely because worrying is physiologically activating. After an employee experiences acute/episodic underperformance, it is reasonable that this employee will worry about his/her work situation and potential consequences. Therefore, perseverative thoughts in the form of rumination or worries are likely proximal outcomes but also mechanisms linking the effects of episodic underperformance on more distal strains.

***Proposition 8**: Acute/episodic underperformance leads to work-related rumination and worries*.

#### Acute/Episodic Underperformance Examples and Distal Outcomes

In most, if not all occupations, employees can experience episodes of underperformance regardless of their general performance level. These episodes vary greatly, from making a split-second aviation error than can cost many people their lives to having a “bad day” as a customer service employee, where many customers leave without their expectations being met. These differences represent the occupation-level consequence of error that will be discussed in detail as a moderator later. To allow for that discussion, it is first important to sample several occupations where acute/episodic underperformance can have different contexts and consequences.

Within the service literature, one prominent form of acute/episodic underperformance is *service failures*, defined as situations where the delivery of service fails to meet customers’ expectations (Holloway and Beatty, 2003). When customers’ expectations regarding the service encounter are unmet, customers perceive injustice and experience negative affective reactions such as rage or anger (McColl-Kennedy et al., 2009). These reactions can then be translated into retaliatory antisocial behavior toward the employee (i.e., customer mistreatment; Groth and Grandey, 2012). This mirrors mistreatment that underperformers receive from other organizational members such as coworkers and supervisors (e.g., Tepper et al., 2011). Therefore, acute/episodic underperforming in a service encounter or a service failure, can result in the experience of a service-related stressor, customer mistreatment. Customer mistreatment can then elicit a range of strain outcomes, some that are the result of ruminating about the events (Wang et al., 2013), while others can be the result of a customer choosing to switch to a different service provider (McColl-Kennedy et al., 2009) and the associated consequences from the supervisor/organization in a monitored climate where “the customer is always right” (Grandey et al., 2004).

A very different occupation that can also be considered a service occupation is the work of police officers. A study examining the link between police officers’ stress and well-being at work revealed that work stress was associated with many well-being outcomes as well as poor coping strategies, such as inappropriately aggressive behavior (Gershon et al., 2002). When a police officer exhibits inappropriately aggressive behavior on the job, this can be considered underperformance and holds a risk of penalty from supervisors and a risk of lawsuits. These in turn are now added stressors for the police officers that can further harm their performance and well-being. Ultimately, stress can result in more aggressive behavior that can then have additional stressful consequences, perpetuating this negative cycle.

Using these two very different occupations, we can see that acute/episodic underperformance can lead both to increased aggressive behavior and to increased exposure to aggression. In both cases, underperformance ultimately results in increased stressors for the employee.

***Proposition 9**: Acute/episodic underperformance can lead to increased aggressive behavior and exposure to aggression*.

*Medical errors* are another form of acute/episodic underperformance. Medical errors are defined as an unintended act, a failure to complete a planned act as intended, or an error in planning, that may cause harm to a patient. Medical errors are typically preventable by the employee and, therefore, constitute acute/episodic underperformance. Stress experiences of medical professionals are often studied as an antecedent of medical errors. For example, studies found greater error rates among employees who experience higher stress, burnout, and depression levels (Williams et al., 2007; Shanafelt et al., 2010), though the cross-sectional designs that were used can only support an association between stress and underperformance, and not any one direction. One study (West et al., 2006) has focused specifically on the well-being consequences of medical errors for the employees (resident physicians) and indeed found increased rates of depression and burnout 3 months later. Another study found that about one third of surgeons whose patients had serious surgical complications experienced traumatic stress in the following month (Pinto et al., 2014). Therefore, medical errors are an example of episodic underperformance that can lead to a wide range of strain and well-being outcomes. Furthermore, medical errors are considered to be a prevalent cause of death, and as such, they are often investigated (Makary and Daniel, 2016). These investigations can lead to additional stress for the employees, as medical errors are often associated with a fear of malpractice lawsuit (Jones et al., 1988). Physicians who make more medical errors are more prone to be sued, ultimately resulting in decreased psychological and physical well-being (Martin et al., 1991; Fileni et al., 2007).

***Proposition 10**: Acute/episodic underperformance can lead to increased risk of lawsuits and associated strains*.

Even in contexts where lawsuits are less likely, acute/episodic underperformance is a stressor and, as such, can result in increased strain and poorer well-being. Within the medical professions, another interesting example for underperformance is evident from the Nursing Stress Scale (Gray-Toft and Anderson, 1981). This earlier work identified many sources of stress for nurses, among them inadequate preparation that includes being unable (due to lack of sufficient training or preparation) to handle aspects of the job that are expected, which can be considered underperformance. This source of stress has been linked to poorer mental health (Lambert et al., 2004; Chang et al., 2006).

Other evidence can be gleaned from the literature on *workplace accidents and injuries*. Accidents, injuries, and cognitive failures can also be considered to be acute/episodic underperformance, as they are often the result of something the employee did, and are associated with work stress (e.g., Wadsworth et al., 2003). Researchers have examined links between stressors and accidents in various occupations. For example, time pressure is associated with transit operators’ accident rates (Greiner et al., 1998), physical and psychological stress are associated with safety behaviors and risk of accidents in construction workers (Leung et al., 2016), and job stress and perceived risk are associated with errors and injuries in offshore petroleum personnel (Rundmo, 1992). While the theoretical link between stress and the ensuing accidents/injuries is relatively well-understood, arguments can be made for the reverse direction, whereby underperformance in the form accidents/injuries can result in subsequent stress. A longitudinal investigation of the effects of underperformance on future stress experiences of employees might reveal that after an episodic underperformance that resulted in an accident or injury, the employees experience greater difficulty meeting the demands of their jobs due to decreased physical ability or to worries regarding a future accident. Therefore, underperformance that results in accidents/injuries can then lead to increased stress experiences and reduced well-being for employees.

***Proposition 11**: Episodic underperformance can lead to decreases in physical and emotional well-being*.

As evident from the reviewed literatures, despite very little research being directly devoted to the negative effects of general and episodic underperformance on subsequent employee health and well-being, these effects are likely substantial. The current paper provides an organizing model for understanding the typical strain responses for acute/episodic versus chronic underperformance and by discussing the interplay between those underperformance types as discussed next.

### The Interplay Between Chronic and Acute/Episodic Underperformance

**Figure 2:**
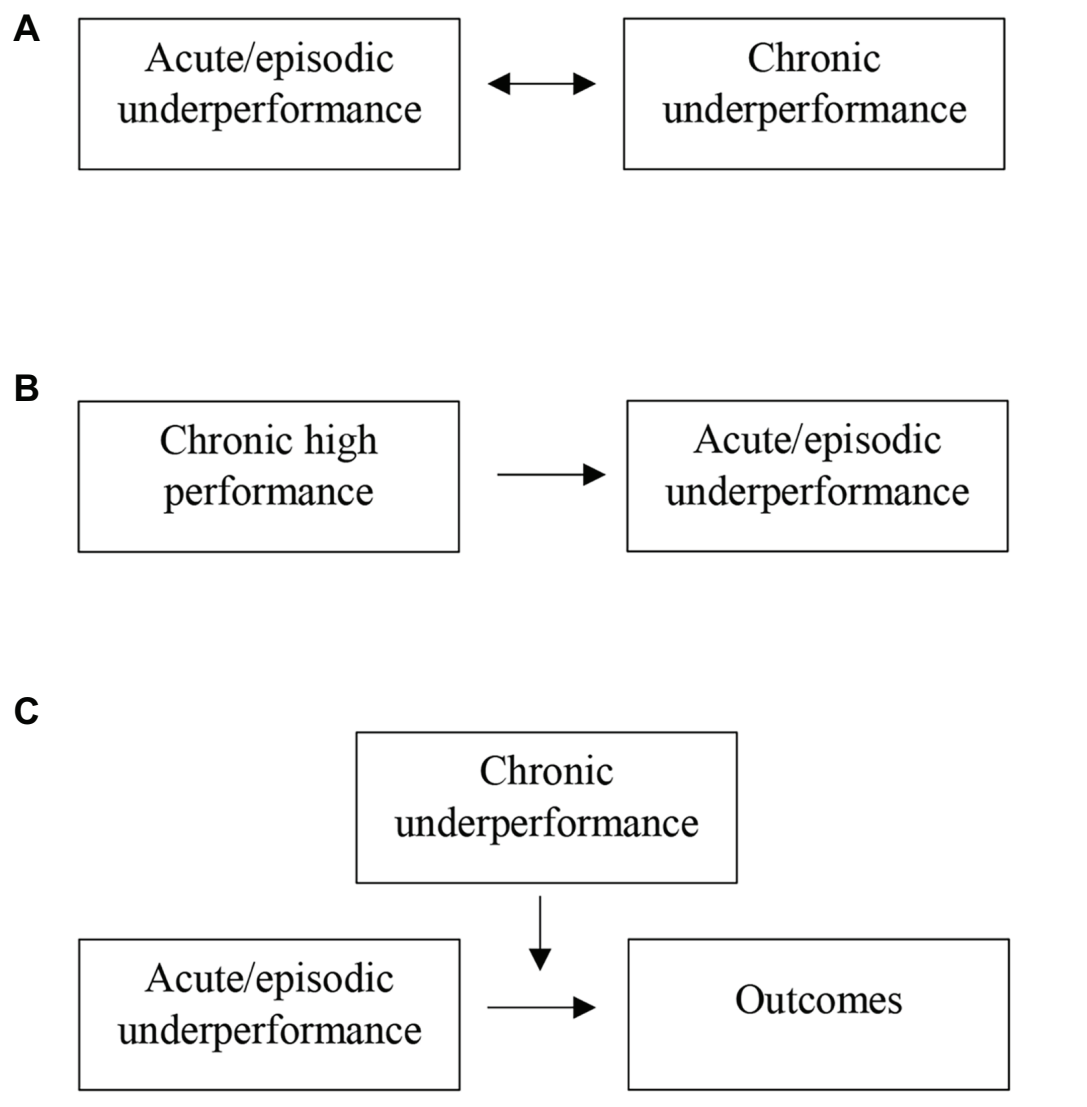
Three potential types of interplay (A-C) between acute/episodic and chronic underperformance.

While there is a clear distinction between what constitutes acute/episodic and chronic underperformance, it is important to note that there is an interplay between the two types, which has some characteristics of a multilevel phenomenon (Bliese and Jex, 2002). There are at least three different ways in which the two types can relate to one another, as depicted in Figure 2. First, many repeated episodes of underperformance can be considered chronic underperformance, and general underperformers may be more likely to make episodic mistakes. This is a simple reciprocal relationship between the two types that reflects two levels of the same phenomenon, within-person and between-person (see Figure 2A).

An exception to the simple reciprocal relationship is the case whereby high performers also get assigned more difficult tasks. These more difficult tasks may hold a higher likelihood of error, such as more complicated cases for surgeons where more things can go wrong or more difficult pieces of computer code to write for software engineers where it is more likely to introduce a bug. This is an interesting phenomenon where high chronic performance could lead to an increased rate of acute/episodic underperformance (see Figure 2B).

Furthermore, there is likely an interaction between the two underperformance types (see Figure 2C). It is thought that employees who are generally high performers are more likely than underperformers to be able to leverage an error as a learning opportunity and not experience subsequent error strain (King and Beehr, 2017). Therefore, the consequences or outcomes for acute/episodic underperformance are likely more severe when the employee is chronically an underperformer. In support of this reasoning, one study revealed that episodic underperforming, specifically, losing in a tennis match, decreased players’ self-efficacy more if they had lower initial levels of self-esteem. This difference was attributed to poorer coping in the form of disengagement and self-blame (Lane et al., 2002).

***Proposition 12**: Chronic and acute/episodic underperformance can affect one another, and chronic performance level can moderate the effects of acute/episodic underperformance*.

### Moderators for Chronic and Acute/Episodic Underperformance

There are many potential theoretical moderators that can affect the relationship between underperformance and its employee outcomes. These theoretical moderators include occupation-level, job-level, and person-level characteristics, as depicted in Figure 1. The moderators that are most relevant to the current conceptualization in each level are described next.

#### Occupational Characteristics

The occupation-level consequence of error is defined by the Occupational Information Network (O*NET) as the seriousness of the result of a mistake made by the employee that was not readily correctable (Peterson et al., 2001). Considering acute/episodic underperforming, the consequences of error vary substantially between occupations, because making a mistake in certain professions (e.g., a nurse or a medical doctor making a medical error) can risk the life of others (in this case, patients), while making a mistake in another profession (e.g., a delivery person mishandling a delivery) will usually only cost time/money. In some cases, it may be unclear how severe the consequences of error are (e.g., a manufacturing employee in a car factory making a manufacturing error can result in life/death consequences or can only be costly in terms of time/money), or there can be variance in the consequences of error within a specific occupation that depend on job-level characteristics. The occupation-level or job-level consequences of error can, therefore, moderate the strain outcomes of acute/episodic underperformance. This would likely be true for a whole range of outcomes, including potential administrative consequences (lawsuits or risk of termination), emotional responses of guilt and anxiety, self-efficacy, and the various well-being outcomes.

***Proposition 13**: Occupation-level consequence of error moderates the effects of underperformance as a stressor*.

#### Job Characteristics

Job characteristics such as organizational constraints and performance pressure are contextual variables that were discussed earlier in this paper. Both can be moderators for the consequences of underperformance, organizational constraints buffering the effects and performance pressure exacerbating them. Organizational constraints are external factors, beyond the employee’s control. It is likely that in the presence of organizational constraints, underperformance will be judged as less severe because it is, at least in part, beyond the employee’s control. Specifically, while objective performance may be significantly lowered in the presence of constraints, supervisor or self-ratings of performance take these constraints into consideration and inflate the performance ratings basing them more on effort than on outcomes (Pindek and Spector, 2016a).

Performance pressure, on the other hand, focuses on the necessity for high performance (Eisenberger and Aselage, 2009). As such, it can be expected that under culture conditions of performance pressure, underperformance will be judged as more severe by both the supervisor and the employee, because the expectation for higher performance highlights the gap between the perceived and ideal performance levels.

Error management culture (e.g., van Dyck et al., 2005) is another potential moderator. Error management culture reflects an environment that enables learning from mistakes and can potentially buffer strain outcomes as well as employment-related outcomes. This is because it pertains to the locus of attention. Locus of attention refers to whether the attention of the individual is directed toward the self or toward the task. If the attention is focused on the task, such as when there is a strong error management culture, then underperformance will more likely result in an increased effort to achieve performance goals. However, if the attention is focused on the self, and specifically on the discrepancy between the perceived and ideal self that is expected under conditions of performance pressure, underperformance will more likely result in negative affective reactions and a depletion of cognitive resources, which will more likely debilitate the individual (Kluger and DeNisi, 1996, 1998). This debilitating effect will likely reduce not only future performance but also the well-being of the employee.

***Proposition 14**: Organizational constraints and error management culture buffer, and performance pressure exacerbates the effects of underperformance as a stressor*.

#### Personal Characteristics

Personal characteristic can arguably moderate the strain consequences of both general and episodic underperformance. Negative affectivity, defined as the dispositional tendency to perceive the world more negatively and experience more negative moods, is a widely studied moderator within the work stress domain (e.g., Penney and Spector, 2005; Zhou et al., 2014). Negative affectivity typically results in an increased likelihood that the employee will appraise a stressor as more severe and that the perceived stressor will lead to greater strain responses. In the case of underperformance, those with high negative affectivity would be more likely to appraise their own lower performance level as severe underperformance and also experience stronger strain responses to it.

Another relevant personality trait is perfectionism or striving for flawlessness and setting unreasonable performance goals. Individuals high on perfectionism are overly critical in their self-evaluations of their own behavior, and this trait is positively linked to stress and burnout (Childs and Stoeber, 2010). Perfectionism has a positive relationship with maximal performance, but it is also associated with lower self-worth, which has a negative effect on typical performance levels, through emotional exhaustion (Hrabluik et al., 2012). When an employee high on perfectionism underperforms, he or she is likely to perceive the underperformance as more severe and have a stronger strain response to it, similar to the effects of negative affectivity in this context.

Another relevant personal characteristic is work locus of control or the degree to which employees believe they have control over their work lives and particularly organizational rewards, as opposed to those being controlled by external forces (Spector, 1988). Having an internal locus of control is associated with positive outcomes, such as higher levels of job satisfaction and perceived support, lower levels of perceived stressors and strains, and higher levels of job performance (Wang et al., 2010). Furthermore, an internal locus of control can buffer the negative effects of many stressors (e.g., Spector, 2011; Sprung and Jex, 2012). It is likely that having an internal locus of control will also buffer the effects of underperformance. This is because underperforming employees who have an internal locus of control will believe that they (rather than the external forces in their work environment) are in control of their performance levels. This will likely lead to extending additional effort in order to improve their performance rather than get anxious or sad about the underperformance that is deemed a misfortune and beyond their control. Work locus of control is particularly relevant to the current framework because it mirrors the idea of locus of attention. That is to say, attention can be focused on the possibility of learning from the mistake and improving future task performance, or it can be on the unchangeably of the situation and on the blow to self-esteem. This focus of attention is affected both by job characteristics such as the error management culture and by personal characteristics such as work locus of control.

Many other moderators of the stressor-strain relationship (e.g., resources; Xanthopoulou et al., 2007, 2009) can be expected to buffer the negative effects of underperformance on strain outcomes, seeing as underperformance is conceptualized as a stressor. However, moderators that are not uniquely relevant to this conceptualization of underperformance as a stressor are not discussed in depth in this paper.

***Proposition 15**: Negative affectivity, perfectionism, and external work locus of control are personal characteristics that exacerbate the effects of underperformance as a stressor*.

One important note is that performance levels are likely part of dynamic relationships with at least some personal characteristics. For example, self-esteem likely buffers the negative effects of underperformance on well-being (Lane et al., 2002), but underperformance may also affect the individual’s self-esteem. Such dynamic processes have been portrayed in the context of self-efficacy spirals and task performance (Lindsley et al., 1995) and are extremely relevant here.

## Discussion

Now that underperformance has been reconceptualized as a stressor, with specific proximal and distal outcomes and moderators, methodological considerations as well as practical implications can be discussed. These considerations can guide researchers who are interested in testing the propositions of this paper or practitioners who wish to apply some of the thinking here to their organizational practices.

### Methodological Considerations

#### Source of Underperformance Ratings

One basic question that underperformance researchers will encounter is whether or not underperformance can be measured objectively. Objective measures of underperformance, when available, would have several advantages. First, when combined with self-reported strain measures, they would result in multi-source data. Multi-source data designs are not employed in most stress studies, but they are desirable because they alleviate concerns that the results reflect common method variance rather than true effects (Spector and Pindek, 2016). Furthermore, objective measures of underperformance, such as when accidents or medical errors are registered, have some additional potential consequences as discussed earlier in this paper. These are the two main advantages of using objective underperformance measures. Unfortunately, the use of objective underperformance ratings is not always possible and very often depends on the specific occupation. This is because objective indices of underperformance do not exist in many occupations. When objective underperformance measures are not possible, then researchers should consider using supervisor-rated underperformance measures. In addition to either objective or supervisor-rated measures, self-rated underperformance should also be assessed, because for many of the described processes, underperformance must be appraised by the employee for the employee reactions to occur. In summary, when designing any study that focuses on underperformance, the source of the rating should always be considered.

#### Sampling

While always true, it is advisable to optimize the sample in light of the specific underperformance research question. For example, an investigation that is focused on error management culture, which is a group level variable (be it work-group or organization), would benefit from a sample that includes many organizations/groups, so that there is sufficient variance on the culture variable. Optimally, a multilevel sample of employees nested within work-groups will be used, whereby employees can report on their group’s error management culture. This would be done using items that refer to the group level culture (e.g., in my work group, it is not common to cover up mistakes) that would later be aggregated to the group level in order to capture the group-level culture. This group-level culture variable can then be modeled as a moderator of the individual-level relationships between underperformance and its outcomes.

If the focus is on the consequence of error, then it would be necessary to sample from heterogeneous occupations. The specific value representing the occupation level consequence of error can be retrieved from O*NET, thus creating a mutli-source design and reducing concerns of common method variance.

For another example, if a study aims to test the moderating role of negative affectivity, then researchers should not focus on a single job, particularly if it is a less attractive job. This is because those jobs could have a disproportional rate of employees high on negative affectivity, in line with the drift hypothesis (Spector et al., 2000).

So far, the examples given all point to the advantages of using heterogeneous samples. However, in some cases using a homogenous sample might allow for more precise measurements. For example, asking specifically about medical errors is clearer than asking generally about errors or underperformance, but it is only relevant in medical jobs. This can also result in limiting the amount of variance that the researchers are not aiming to explain (error variance), thus contributing to clearer result patterns. In summary, the variables included in the study should be considered when choosing the sample for the study.

#### Time

Many of the processes described in this paper were supported by findings from cross-sectional designs. While cross-sectional data can be useful for many different purposes (Spector, 2019), cross-sectional designs would not generally be advisable for researchers examining underperformance as a stressor. This is because stressors and strains have an established role as antecedents of subsequent decreases in performance, and cross-sectional findings of links between stress variables and underperformance would more readily be attributed to the direction theorized in the stress-performance framework (Jex, 1998; Bliese et al., 2017).

Therefore, investigations into the effects of underperformance on subsequent decrements to well-being would benefit greatly from the use of longitudinal designs. Using such designs, researchers could examine the change in well-being indicators, comparing the levels prior to an episodic underperformance to the levels after an error had been made. With chronic underperformance, it is harder to sequence the measurements over time. Nevertheless, meaningful before-after comparisons could be aligned with the periodic performance appraisals that are customary in many organizations. One particularly interesting research opportunity is timing the research to be around the first formal performance appraisal that an employee receives on a certain job, as has been done with other organizational phenomena (e.g., Spector et al., 2015). Alternatively, cross-lagged panel designs could be used to model both directions of effects: stress leading to subsequent underperformance and underperformance leading to subsequent stress.

A related point is the duration of effects of underperformance. As with most stressors, we do not know how long the effects of underperformance can be expected to last, but examining different lags (e.g., Meier and Spector, 2013) can prove beneficial to future researchers designing their studies. Another unknown is whether those effects manifest immediately after the first instance of underperformance, or if repeated episodes are required before the more distal strain effects occur (Dormann and van de Ven, 2014). These factors likely depend on the type of job, severity of underperformance, and whether or not there were administrative consequences such as a lawsuit of termination. Longitudinal designs with different time lags can uncover these temporal characteristics of the effects of underperformance.

One important design type that is particularly relevant for capturing episodic underperformance is the diary design, or experience sampling methodology. These designs require that the employee completes the same set of questions referring to a limited time frame, for example, questions that repeat every day and refer to that specific day or questions that the employee has to fill out only after a certain acute event has occurred. The repeated measures allow researchers to separate out the average level from the day level (Bolger and Laurenceau, 2013). Therefore, an individual, experiencing a low-performance day in comparison to that individual’s own average performance level would be an approximation for episodic underperformance. The chronic performance levels can be acquired by using separate questions that reference the person-level. While seemingly a straightforward way of capturing episodic underperformance, one notable problem is that in some occupations, arguably the ones where the consequences of error are more severe, it is possible that mistakes that cannot easily be rectified are relatively rare. In such cases, it would be difficult to conduct an experience sampling study over a limited time period because there might not be enough cases of episodic underperformance to allow for any meaningful analyses. This problem could perhaps be addressed by assessing near misses and not just errors, as they may share at least some outcomes. Another limitation of using a diary design to study underperformance is that it does not provide information on the long term effects of this underperformance.

In summary, studies focusing on underperformance as a stressor need to consider several methodological issues, including sources of the data, sampling of participants, and timing of the measurements. These considerations are not unique to underperformance (Spector and Pindek, 2016), but the unique aspects that stem from researching specific questions within this framework are crucial for the ability to conduct quality research on the topic.

### Practical Implications

How should organizations deal with underperformance? Organizations have traditionally used performance appraisals to decide which employees to promote and which ones to layoff. However, once the underperformance is uncovered either by locating the underperforming employees or by the typical acute/episodic underperformance manifestations in the organization, more analyses can reveal the causes of these underperformance phenomena. Different causes would lead to different recommendations. For example, if the underperformance resulted from skill issues, then providing training may resolve the problem. If the cause is low motivation, then perhaps changes to the job design can be implemented. If chronic underperformance is the result of a poor person-job fit, then perhaps re-skilling and helping employees make career adjustments within the organization could resolve the problem. Job termination should not be considered the best solution in most cases.

In this paper, we have discussed the negative consequences of underperformance for the employees themselves, and not for the organizations. Therefore, one could arguably surmise that it is better for the employees, and not just for the organizations, to remove underperformers from the job, seeing as their underperformance was hurting their well-being. This is not, however, the takeaway message from this paper. Terminating employment is, after all, considered detrimental to the individual’s well-being and health, as it is even associated with a reduced life expectancy (Sullivan and von Wachter, 2009). Typically, laying off an underperforming employee would not be showing them kindness.

However, this conceptualization of underperformance as a source of stress for the employees demonstrates the damage to employee’s well-being and, depending on the consequence of error, underperformance can have serious ramification for society at large. Therefore, keeping underperforming employees on the job as a mercy is ill-advised as well. Rather, organizations should tailor the responses to the causes of underperformance, benefitting both the employees and the organization. Examples include the already discussed error management culture that can help employees make fewer episodic mistakes in the future, as well as help them cope better with the emotional outcomes of those mistakes. It would seem that laying off underperforming employees is the “easy way out” for organizations, but seeing as underperformance is detrimental to both the organizations and the employees themselves, there are likely alternative courses of action that can benefit both organizations and employees.

### Conclusion

This paper’s main focus is on re-conceptualizing underperformance as a stressor that threatens employees’ well-being. Specifically, acute/episodic and chronic are two distinct types of underperformance. These two types of underperformance share some but not all outcomes and moderators. Helping employees reduce and cope with their underperformance is beneficial for employees and organizations alike.

## Data Availability Statement

The original contributions presented in the study are included in the article/supplementary material; further inquiries can be directed to the corresponding author.

## Author Contributions

The author confirms being the sole contributor of this work and has approved it for publication.

## Acknowledgments

I thank Paul Spector for his helpful comments on an earlier version of this paper.

## Conflict of Interest

The author declares that the research was conducted in the absence of any commercial or financial relationships that could be construed as a potential conflict of interest.
